# Circulating Extracellular RNA Markers of Liver Regeneration

**DOI:** 10.1371/journal.pone.0155888

**Published:** 2016-07-14

**Authors:** Irene K. Yan, Xue Wang, Yan W. Asmann, Hiroaki Haga, Tushar Patel

**Affiliations:** 1 Department of Transplantation, Mayo Clinic, 4500 San Pablo Road South, Jacksonville, FL 32224, United States of America; 2 Department of Cancer Biology, 4500 San Pablo Road South, Jacksonville, FL 32224, United States of America; 3 Department of Health Sciences Research, 4500 San Pablo Road South, Jacksonville, FL 32224, United States of America; SAINT LOUIS UNIVERSITY, UNITED STATES

## Abstract

**Background and Aims:**

Although a key determinant of hepatic recovery after injury is active liver regeneration, the ability to detect ongoing regeneration is lacking. The restoration of liver mass after hepatectomy involves systemic changes with coordinated changes in gene expression guiding regenerative responses, activation of progenitor cells, and proliferation of quiescent hepatocytes. We postulated that these responses involve intercellular communication involving extracellular RNA and that these could represent biomarkers of active regenerative responses.

**Methods:**

RNA sequencing was performed to identify temporal changes in serum extracellular non-coding RNA after partial hepatectomy in C57BL/6 male mice. Tissue expression of selected RNA was performed by microarray analysis and validated using qRT-PCR. Digital PCR was used to detect and quantify serum expression of selected RNA.

**Results:**

A peak increase in extracellular RNA content occurred six hours after hepatectomy. RNA sequencing identified alterations in several small non-coding RNA including known and novel microRNAs, snoRNAs, tRNA, antisense and repeat elements after partial hepatectomy. Combinatorial effects and network analyses identified signal regulation, protein complex assembly, and signal transduction as the most common biological processes targeted by miRNA that altered. miR-1A and miR-181 were most significantly altered microRNA in both serum and in hepatic tissues, and their presence in serum was quantitated using digital PCR.

**Conclusions:**

Extracellular RNA selectively enriched during acute regeneration can be detected within serum and represent biomarkers of ongoing liver regeneration in mice. The ability to detect ongoing active regeneration would improve the assessment of hepatic recovery from liver injury.

## Introduction

Although the liver has the capability to regenerate following injury, the mechanisms of liver regeneration remain incompletely understood. Liver regeneration involves activation of progenitor cell populations recapitulating signaling mechanisms involved in liver development.[1] These result in the restoration of liver mass through a highly-coordinated proliferation of most of the pre-existing quiescent hepatocytes in the remnant, liver. Systemic responses contributing to hepatic regeneration can be experimentally studied after partial hepatectomy or liver resection.[2] Following partial hepatectomy, rapid changes in gene expression occur in a sequential and coordinated manner. To date, studies of the alterations in gene expression that occur after partial hepatectomy have mostly focused on the detection of tissue mRNAs or non-coding RNAs (ncRNAs), such as microRNAs (miRNA).[3–7] However, these comprise a very small fraction of transcribed genes. However, genomic studies have provided evidence of pervasive transcription of a large number of other ncRNA genes such as long non-coding RNA (lncRNA), although the biological functions for many of these are unknown.

Many cells, including progenitor and differentiated human liver cells, can release extracellular RNA (exRNA) enclosed within extracellular vesicles (EVs) into their local environment. The release of exRNA occurs in a selective manner because there are significant differences between the RNA content of the vesicles and that of their cells of origin. We and others have identified that the majority of the RNA content found in liver cell-derived EVs comprises small RNA (< 200 bp) and ncRNA.[8] The transfer of this RNA across cells through EV release and uptake can serve as a potent mechanism by which inter-cellular communication occurs.[9] We have shown that cell-to-cell transfer of functionally-active ncRNA within EVs can modulate gene expression and activate the signaling pathways involved in cell proliferation and apoptosis.[10] Several other biological functions have been shown to be modulated by vesicular release and uptake. On this basis, we propose that changes in exRNA can serve as a mechanism to enable genetic reprogramming of quiescent hepatocytes and induce cell proliferation during hepatic regeneration and moreover that the transfer of exRNA could serve as a potent signaling mechanism that coordinates regenerative responses.

The exRNA that are released into the circulation during hepatic regeneration also have the potential to serve as specific biomarkers of the hepatic regenerative response. Their presence within circulating EV or their association with proteins or lipoproteins may protect these RNA from degradation. Recent studies have shown that EV can be detected in the circulation following liver injury.[8] Moreover, selective alterations in circulating miRNA have also been reported in liver injury.[11] RNA sequencing studies have identified many different types of RNA within EV in the circulation. We therefore sought to identify exRNA released during the early phases of liver regeneration as candidate markers of ongoing active regeneration. We used an unbiased and comprehensive RNA sequencing-based profiling strategy to identify circulating exRNA following partial hepatectomy. These studies identified several non-coding RNA in the circulation during liver regeneration that could be useful biomarkers of the regenerative response.

## Materials and Methods

### Isolation of circulating or tissue RNA

Twelve-week-old C57BL/6 mice (Charles River Laboratory) were subjected to a partial hepatectomy during which the left and medial hepatic lobes were ligated and excised, resulting in the removal of 65–70% (2/3 partial hepatectomy) of the liver. For detection of circulating exRNA, serum samples were obtained by cardiac puncture from four mice each at 1, 6 and 24 hours after partial hepatectomy. The entire surgical process was approved by the Charles River Institutional Animal Care and Use Committee. Details of methods to minimize potential suffering, including surgical anesthesia and postsurgical pain relief and supportive care, can be requested from Charles River Laboratories (Wilmington, MA). RNA was isolated using ExoQuick (Systems Biosciences, Mountain View, CA). The exRNA content was purified using SeraMir kits (Systems Biosciences, Mountain View, CA) and quantified using a Nanodrop ND-1000 (NanoDrop Technologies, Wilmington, DE). For tissue RNA studies, mice were euthanized, and the residual liver tissue was harvested at 15, 45, 90 and 240 minutes posthepatectomy; tissue samples were obtained from five animals at each time point. A half of each liver was stored in RNA-later solution (Invitrogen, Carlsbad, CA), and the rest was either lysed for RNA isolation or was immediately frozen on dry ice and subsequently stored at -80°C. Total RNA was isolated with Trizol (Invitrogen, Carlsbad, CA) according to the manufacturer's instructions. We considered changes occurring within the first 6 hours as indicating early stages of hepatic regeneration after partial hepatectomy.

### RNA sequencing and analysis

Purified exRNA was made into Illumina Tru-Seq RNA-Sequencing libraries, and samples were indexed using the NEBNext Small RNA Library prep kit (Illumina, San Diego, CA). Libraries were amplified for 12–18 cycles according to the manufacturer’s instructions. Amplified libraries were sized-fractionated using Sage Bioscience Pippin prep station on 2% agarose gels (Sage Bio cat # SDF2010) with a collection range of 135 bp—200 bp. The size and concentration of the purified libraries were analyzed using an Agilent 2100 Bioanalyzer high sensitivity DNA chip. Libraries were pooled in equal molar amounts for multiplex sequencing on an Illumina MiSeq sequencer.

### Bioinformatics analysis

Sequencing analysis was conducted using the Exosome/Small RNA-seq Analysis Kit 1.0 (Maverix Biomics, San Matteo, CA). Raw sequencing reads with a length of 75 bp were quality checked for potential sequencing issues and contaminants using FastQC (Babraham Institute, version 0.10.1). Adapter sequences, primers, poly-Ns, and bases with quality scores below 28 were trimmed using fastq-mcf of ea-utils and PRINSEQ.[12, 13] Reads with a remaining length of fewer than 16 bp after trimming were discarded. Pseudo single-end reads were formed by merging the post-trimmed paired-end reads using SeqPrep and were mapped to the mouse genome (mm10) using Bowtie.[14] Read coverage on forward and reverse strands was computed using SAMtools and BEDtools.[15–17] Raw read counts were calculated for known gene categories, including ncRNAs, antisense transcripts, coding and intronic regions of mRNAs, and repeats. The raw read counts per loci across samples were normalized, and differential gene expression between time points and baseline controls were analyzed using DEseq.[18] Significantly and differentially-expressed exRNA genes were determined using adjusted P-values of ≤ 0.05. Hierarchical clustering of the normalized gene expression values from the control sample and each time point were carried out using Pearson correlation distance. Additional sequencing analysis was also performed using the miRNA analytical workflow CAP-miRSeq, developed at the Mayo Clinic Bioinformatics Core, and by using the Genboree workbench exceRpt pipeline from the NIH Extracellular RNA Communications Consortium.[19] Sequencing data will be made available at the exRNA Atlas.[20] miRNA target analysis was performed using the MIRROR algorithm (v. 2.0), which comprehensively integrates data from several target prediction programs.[21] Gene ontology analysis was performed using the Database for Annotation, Visualization and Integrated Discovery DAVID v 6.7.[22, 23] Network analysis to identify interactions amongst putative target genes was performed using the String program (v. 9.05).

### Microarray Analysis

Five micrograms of total RNA obtained from livers at different time-points up to 240 minutes after partial hepatectomy were hybridized onto custom miRNA microarray chips (OSU_CCC version 3.0), containing approximately 1100 miRNA probes, including 345 human and 249 mouse miRNA genes as well as predicted and ultraconserved RNA genes, with each gene spotted in duplicates. The miRNA microarray is based on a one-channel system. RNA labeling and hybridization on miRNA microarray chips were completed as described.[24] Hybridization signals were detected using a Perkin-Elmer ScanArray XL5K. Quantarray software (Perkin-Elmer, Wellesley, MA) was used to quantify scanner images. Raw data were normalized and analyzed using the Statistics for Microarray Analysis R package from the Speed Berkeley Research Group.

### Real-Time PCR Analysis

Total RNA was isolated from frozen liver specimens using Trizol (Invitrogen, Carlsbad, CA). Expression levels of specific mature miRNAs were confirmed by real-time PCR analysis using a TaqMan Human MicroRNA Assay kit (Applied Biosystems, Foster City, CA).

### Digital PCR

Digital PCR was performed using the QX100 system (Bio-Rad, Hercules, CA). cDNA was transcribed from RNA treated with RNase-free DNase I (Qiagen, Maryland, MD) and an RNase inhibitor (10U/μl) (Invitrogen, Carlsbad, CA) using iScript cDNA synthesis kit (BioRad, Hercules, CA). Reaction mixtures were prepared using 10 μl ddPCR 2x Master Mix (Bio-Rad, Hercules, CA), 1 μl 20x Primer & TaqMan Probe Mix (Applied Biosystem, Foster City, CA), 5 μl nuclease free water, and 4 μl reverse-transcribed product. 1 nL reaction droplets were generated using a QX100 Droplet generator (Bio-Rad, Hercules, CA) and a droplet generator DG8 cartridge (Bio-Rad, Hercules, CA) containing 20 μl of reaction mixture and 70 μl of droplet generation oil (Bio-Rad, Hercules, CA) per well. Thirty-five microliters of the generated droplets were transferred to 96-well PCR plates and amplified using a thermal cycler as follows: initial denaturation at 95°C for 10 minutes, followed by 40 cycles of 94°C for 30 seconds and 60°C for 1 minute, and finally 98°C for 10 minutes. The fluorescence intensity of each droplet was then measured using the QX100 droplet reader (Bio-Rad, Hercules, CA). Data was analyzed using the QuantaSoft software (Version 1.3.2.0, Bio-Rad, Hercules, CA) with thresholds based on fluorescence of negative controls.

### Statistical analysis

Data were expressed as the mean and standard error from four biological replicates for RNA sequencing and digital PCR assays and from three replicates for microarray analysis at each time point. Comparisons between groups were performed using the two-tailed Student’s t test. Results were considered to be statistically significant when P < 0.05.

All authors had access to the study data and had reviewed and approved the final manuscript

## Results

### Serum exRNA is increased after hepatectomy

**Fig 1:**
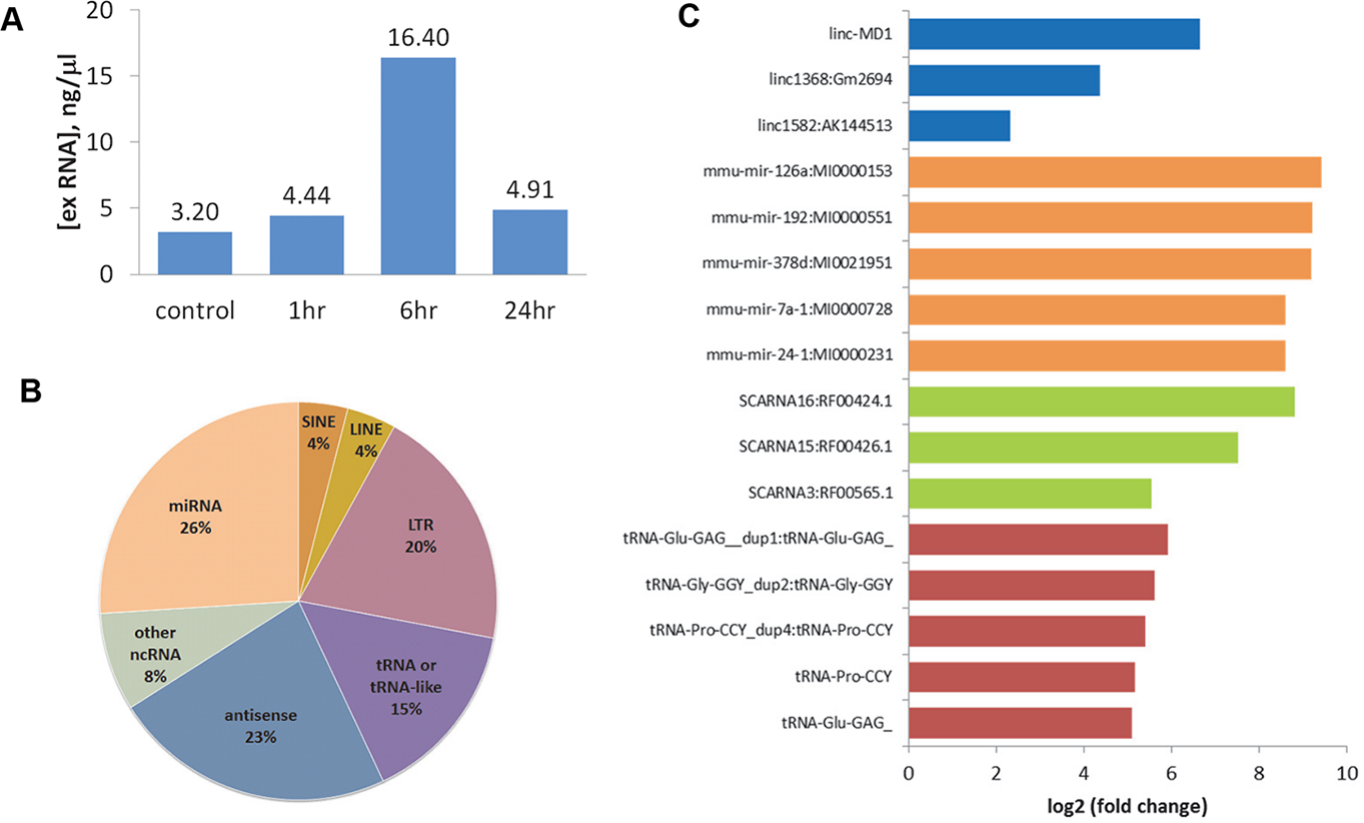
Circulating extracellular RNA (exRNA) after partial hepatectomy. (A). Serum was collected from mice at the indicated time points after partial hepatectomy, and exRNA was isolated. Total exRNA was quantitated using NanoDrop. An increase in circulating total exRNA was seen at six hours after partial hepatectomy. Bars represent average concentration of total exRNA yield from 4 separate samples. (B and C) RNA seq was performed on serum exRNA, and analysis of the serum exRNA was performed using the Maverix pipeline. Differentially-expressed extracellular non-coding RNA were determined across samples. (B) Non-coding RNA that are mapped include miRNA, tRNA, snoRNAs, antisense transcripts and repeat elements. (C). Bar chart representing the most highly differentially-expressed circulating EV non-coding RNA within selected classes six hours after partial hepatectomy. These differentially-expressed extracellular non-coding RNA are candidate biomarkers of a liver regenerative response.

There was a transient increase in circulating exRNA following partial hepatectomy (Fig 1A), with a five-fold increase noted six hours after hepatectomy. exRNA was isolated at selected time points for RNA sequencing, and downstream analyses was performed to identify alterations in non-coding RNA that occurred during hepatic regeneration. Sequencing analyses identified several small non-coding RNAs, including microRNAs, snoRNAs, tRNA, antisense, and repeat elements that were significantly and differentially expressed after partial hepatectomy (Fig 1B). The most significantly-altered, differentially-expressed circulating extracellular non-coding RNAs in selected classes are shown in Fig 1C. These represent candidate circulating non-coding RNA biomarkers of the hepatic regenerative response although, with the exception of miRNAs, their biological functions are not well known.

### Selective alteration in circulating miRNAs during regeneration

**Fig 2:**
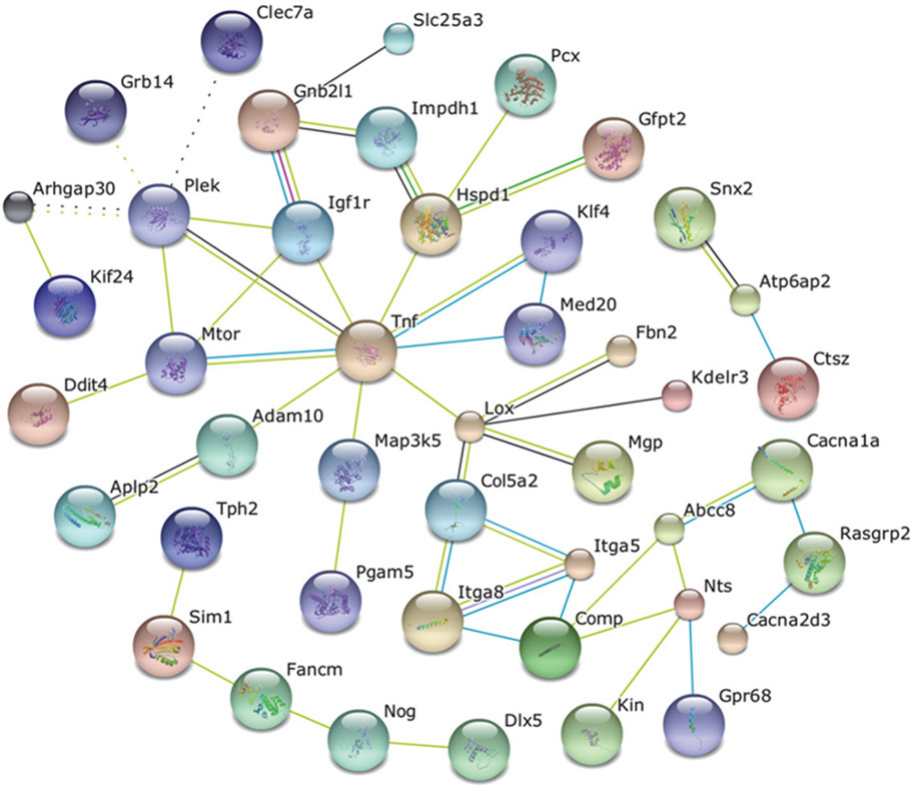
Bioinformatics network analysis of miRNA target genes. The target genes of the 52 miRNAs significantly altered in the circulation after partial hepatectomy were analyzed using the miRror program. Three hundred and five genes were predicted, and network analysis of these genes using String 9.05 indicated interactions amongst several identified targets.

miRNAs have been the most extensively studied non-coding RNAs, and alterations in miRNA expression have been reported following partial hepatectomy.[5, 7] First, we analyzed the miRNA that were significantly altered in serum after partial hepatectomy and hence candidate signaling mediators involved in the regenerative response (Table 1). A combinatorial approach that comprehensively integrates data from several target prediction programs was used to identify putative targets of this set of 52 miRNA. This analysis identified 305 targets that could be altered as a result of exposure to circulating exRNA (S2 Table). Gene ontology analysis identified that the most common biological processes that would be targeted were signal regulation, protein complex assembly, and signal transduction (Table 2). Network analysis to identify interactions amongst these putative targets revealed five interaction nodes with 3 or more interactions, and these included mammalian target of rapamycin, insulin-like growth factor 1 receptor, heat shock 60kd protein 1, lysyl oxidase, integrin-alpha 8, neurotensin, and tumor-necrosis factor (Fig 2). Future studies to identify the contribution of these genes in specific biological processes or systemic responses associated with hepatic regeneration are warranted.

**Table 1:** Extracellular miRNA that are significantly altered in expression following partial hepatectomy.

miRNA	1 hour	6 hours	24 hours
mmu-let-7f-1:MI0000562	-0.1404	4.4985	0.1601
mmu-let-7f-2:MI0000563	-0.0952	4.5235	0.1427
mmu-let-7f-5p:MIMAT0000525_1	-0.0508	4.5536	0.1601
mmu-let-7i:MI0000138	-0.0850	3.7451	0.3100
mmu-let-7j:MI0021908	-0.4258	5.2759	0.2802
mmu-mir-100:MI0000692	2.4487	7.3276	2.7052
mmu-miR-100-5p:MIMAT0000655	2.4487	7.3276	2.7052
mmu-mir-126a:MI0000153	5.1592	9.4294	5.1601
mmu-mir-139:MI0000693	0.3815	6.3969	3.2395
mmu-miR-139-5p:MIMAT0000656	-0.8408	6.2850	3.0171
mmu-mir-140:MI0000165	0.5319	4.6139	1.0748
mmu-mir-143:MI0000257	3.2164	6.4408	2.6927
mmu-miR-143-3p:MIMAT0000247	3.1881	6.4337	2.6674
mmu-mir-152:MI0000174	1.7441	7.9609	3.1046
mmu-mir-1843a:MI0004155	0.4222	5.6430	3.0875
mmu-mir-192:MI0000551	2.9194	9.2163	4.8788
mmu-miR-192-5p:MIMAT0000517	2.9194	9.2159	4.8788
mmu-mir-199a-1:MI0000241	0.7066	5.9376	0.1912
mmu-mir-199a-2:MI0000713	0.7066	5.9376	0.1912
mmu-miR-199a-3p:MIMAT0000230_1	1.1592	6.5665	0.6801
mmu-mir-199b:MI0000714	1.2746	6.5731	0.6801
mmu-mir-22:MI0000570	1.6906	7.4175	3.5305
mmu-mir-24-1:MI0000231	3.9802	8.5986	4.0989
mmu-mir-24-2:MI0000572	3.4259	8.0358	3.5615
mmu-mir-25:MI0000689	0.6215	3.8406	1.1102
mmu-miR-25-3p:MIMAT0000652	0.6215	3.8371	1.1102
mmu-mir-26a-1:MI0000573	0.6152	4.2731	-2.6833
mmu-mir-26a-2:MI0000706	0.6152	4.2764	-2.6833
mmu-miR-26a-5p:MIMAT0000533	0.6152	4.2731	-2.6833
mmu-mir-27b:MI0000142	1.6244	6.1999	2.0491
mmu-mir-29a:MI0000576	1.2466	8.3073	4.4929
mmu-miR-29a-3p:MIMAT0000535	1.2466	8.3055	4.4929
mmu-mir-30d:MI0000549	1.1411	6.0601	1.6790
mmu-mir-375:MI0000792	-0.0108	6.7202	2.6546
mmu-miR-375-3p:MIMAT0000739	-0.0108	6.7202	2.6546
mmu-mir-378a:MI0000795	2.7872	8.3303	3.5764
mmu-miR-378a-3p:MIMAT0003151	2.7405	8.3261	3.5652
mmu-mir-378d:MI0021951	3.6186	9.1961	3.8916
mmu-mir-451a:MI0001730	0.8335	3.3382	1.7719
mmu-mir-532:MI0003206	2.2244	6.3535	2.3915
mmu-mir-7a-1:MI0000728	3.1592	8.6006	5.4766
mmu-mir-7a-2:MI0000729	3.1592	8.5931	5.4766
mmu-miR-7a-5p:MIMAT0000677_1	3.1592	8.5931	5.4766
mmu-mir-92a-2:MI0000580	0.6495	4.7760	3.2723
mmu-miR-92a-3p:MIMAT0000539_1	0.6495	4.7760	3.2723
mmu-mir-99a:MI0000146	2.8938	7.6855	3.3968
mmu-miR-99a-5p:MIMAT0000131	2.8938	7.6855	3.3968
mmu-mir-99b:MI0000147	1.8221	6.4291	2.4322
mmu-miR-99b-5p:MIMAT0000132	1.8221	6.3610	2.2735

Differentially-expressed exRNA genes were selected using adjusted P-values of ≤ 0.05. The Log2-fold change in miRNA expression compared with baseline is reported at the indicated time points after partial hepatectomy.

**Table 2:** Functional gene annotation of putative exRNA targets.

Gene Ontology Biological Process	Fold Enrichment	False discovery rate	P-Value (x10^-3^)	Bonferroni (x10^-3^)
Extracellular structure organization	5.0	0.1	0.065	0.092
Embryonic organ morphogenesis	4.2	0.97	0.59	0.58
Extracellular matrix organization	5.4	1.1	0.68	0.64
Embryonic organ development	3.1	4.6	2.8	0.99
Collagen fibril organization	13.0	5.4	3.3	0.99

Predicted targets of circulating exRNA that are significantly altered after partial hepatectomy were identified using the mirror algorithm, and functional annotation with gene ontology was performed to identify the most common biological processes modulated by these targets using DAVID.

**Fig 3:**
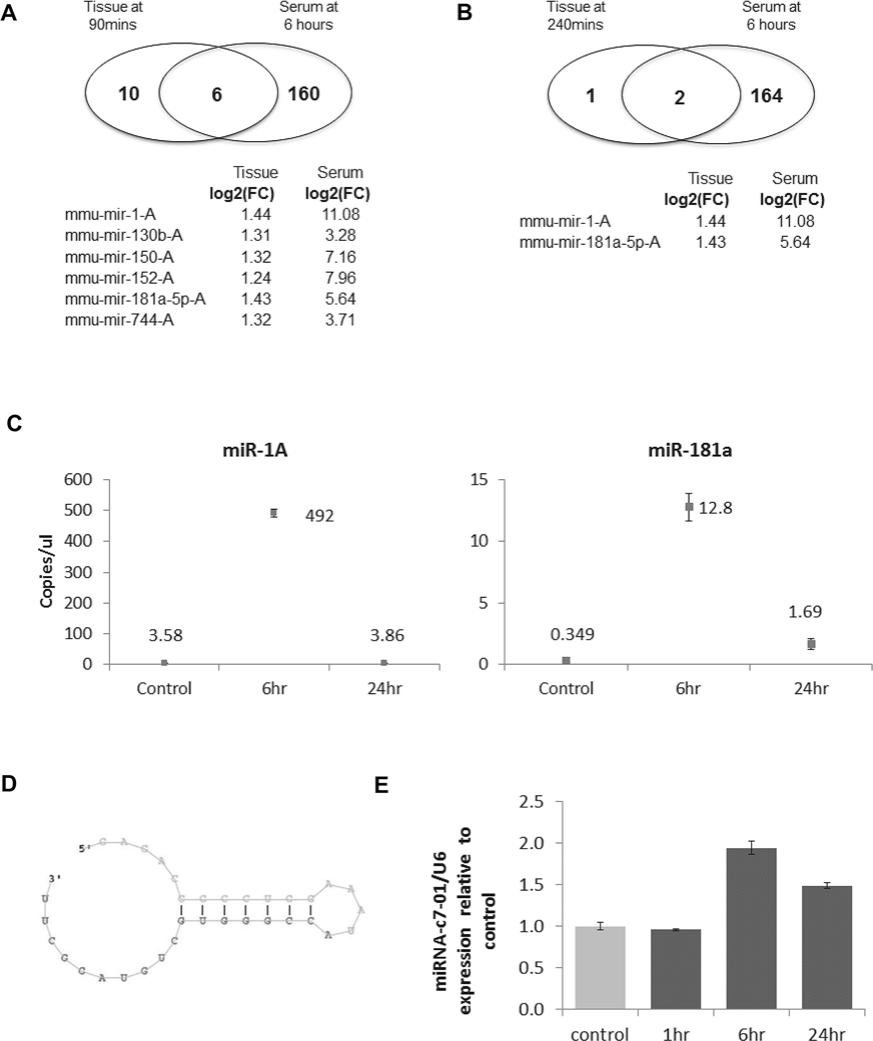
Circulating extracellular RNA after partial hepatectomy. (A and B) Comparison with tissue expression. miRNA expression was determined in liver tissues obtained at (A) 90 minutes and (B) 240 minutes after partial hepatectomy and were compared with miRNA detected in the serum six hours after partial hepatectomy. The Venn diagrams indicate miRNAs that had over 1.2 log-2 fold increase in both liver tissue and serum compared to baseline levels. The miRNA with the greatest increase in expression in both tissue and serum are indicated, with an increase in miR-1-A and miR 181a-5pA noted in hepatic tissue at both time points. (C) Quantitation by droplet digital PCR. A droplet digital PCR assay was used to analyze the expression of miR-1A, miR-181a-5p-A, and miR-222 in serum obtained at baseline, 6, and 24 hours after partial hepatectomy. exRNA was isolated from serum samples at each time point, treated with DNase, and reverse transcribed to obtain cDNA, and of which, 2ul was then emulsified into 1nL reaction droplets prior to target DNA amplification (40 cycles) performed using TaqMan miRNA assays. The fluorescence amplitude of each droplet was measured, and the target concentration (copies/ul) was calculated. No copies of miR-222 were detected. Data for other miRNAs represent merged data of mean quantitation from four technical replicates of one sample at each time point, with error bars showing the 95% confidence intervals after statistical analysis of positive and negative reactions based on poisson distribution. (D and E) Novel circulating extracellular miRNA. D. Putative miRNA with a genomic location mapped to chromosome 7 (pmiR-c7-01) identified by bioinformatics analysis of RNA sequencing data of circulating exRNA after partial hepatectomy. E. Serum expression of pmiR-c7-01 by qRT-PCR using custom- designed specific primers at indicated time points after partial hepatectomy.

We postulated that changes in circulating exRNAs would reflect alterations in hepatic RNA during hepatic regeneration. In this model, portions of the liver that undergo injury are completely removed, and thus subsequent changes are unlikely to reflect hepatic injury. However, circulating exRNA could also reflect non-regeneration related changes, such as non-specific release from other injured cells or local or systemic changes related to anaesthesia or the surgical procedure. To further determine the relevance of changes in circulating exRNA, we examined tissue expression of miRNA at very early stages of regeneration after partial hepatectomy. Changes in miRNA expression have been reported to occur during regeneration after partial hepatectomy, but most studies have described changes that occur several hours after hepatectomy. Although the expression of most miRNAs did not change, we observed early alterations in hepatic expression of ~10% of miRNA by >5 fold (P < 0.05) after partial hepatectomy. The expression levels of seven selected miRNAs from five different time points were validated by RT-PCR, and in all cases, the expression levels observed paralleled the data obtained from the microarray analysis (data not shown). To detect the earliest changes in tissue expression, we focused our efforts on those miRNA that had deregulated expression within an hour after partial hepatectomy. Sixteen of the 496 miRNA identified had expression that was a log2-fold increase of 1.2 or more at 90 minutes after partial hepatectomy. Of these 16, three were also increased in livers collected after 240 minutes, indicating a sustained alteration in tissue expression. A comparison of miRNA that were increased within hepatic tissues as well as elevated in the circulation following partial hepatectomy revealed that the most prominent and sustained changes occurred for miRNA-1A and miRNA-181a-5p-A (Fig 3A and 3B). Both of these were elevated after partial hepatectomy in liver tissues at 90 and 240 minutes and were found elevated in serum after six hours.

### Extracellular miRNA can be detected and quantitated using digital PCR

For validation, the expression of miR-1A and miR-181 was examined in exRNA obtained from serum samples obtained from an independent group of mice undergoing partial hepatectomy. The expression of these two miRNA was barely detectable by qRT-PCR, with cycle threshold (Ct) values > 40 for baseline and greater than 35 at the assayed time points after partial hepatectomy. To detect and quantitatively assay these circulating RNA transcripts with adequate sensitivity, we used a digital PCR assay.[25] In our droplet digital PCR analysis, droplets containing the target transcript had increased fluorescence following amplification, whereas droplets lacking the target remained at baseline levels of fluorescence. The droplet reader software counts positive and negative droplets based on a threshold of fluorescence between well-defined populations of high and low fluorescence amplitude droplets. The expression of miR-1A and miR-181 was detected at baseline and was increased at six and 24 hours after hepatectomy (Fig 3C). In contrast, digital PCR analysis did not detect any miR-222, a miRNA that is increased in tissue samples at six hours after partial hepatectomy but was not detected in the circulation in our RNA sequencing studies.

### Extracellular non-coding RNA as biomarkers

Non-coding RNA other than known miRNA could have potential value as markers. We have shown that lncRNA can be detected within EV released from both malignant and non-malignant hepatocytes.[26–28] Therefore, we analyzed the expression of lncRNA. Although the relative abundance of lncRNA versus miRNA was not possible to determine with our library preparation protocol, which selected for smaller ncRNA, we identified a few lncRNA that were enriched in the circulation following partial hepatectomy (Table 3). Next, we sought to identify novel differentially expressed miRNA candidates from our RNA sequencing data. To do this, we used the CAP-miRSeq pipeline to identify candidates on the basis of secondary structural predictions. The most highly differentially expressed candidate was a 34-nucleotide RNA fragment with a hairpin loop but no identifiable sequence homology to known genes. The genomic location mapped to chromosome 7, and we designated this novel RNA as chromosome 7-putative miRNA 01 (pmiR-c7-01) (Fig 3D). To validate the results, we designed specific primers to detect pmiR-c7-01 and analyzed its expression in the serum expression using qRT-PCR (Fig 3E). Consistent with the data from the RNA sequencing studies, we identified temporal changes in expression of this novel miRNA following partial hepatectomy. Although their biological roles during hepatic regeneration are not known, these and other significantly altered ncRNA may also have the potential for use as biomarkers of active hepatic regeneration.

**Table 3:** Circulating extracellular long non-coding RNA.

	Log2 Fold Change		
lncRNA	1 hr	6 hrs	24 hrs
linc-MD1	0.53	6.64	1.82
linc1368:Gm2694	1.92	4.36	1.92
linc1582:AK144513	2.59	2.32	0.35
AK082072	2.23	1.70	0.47
lincRNA-Sox2:BC051220	0.82	1.35	0.11
lincRNA-p21:HM210889	1.09	0.96	0.39
linc1609:AK144624	0.16	1.48	0.02

Extracellular long non-coding RNAs (lncRNA) detected in serum at indicated times following partial hepatectomy; log2-fold change compared to baseline controls.

### Bioinformatics analyses for discovery of Extracellular RNA

**Fig 4:**
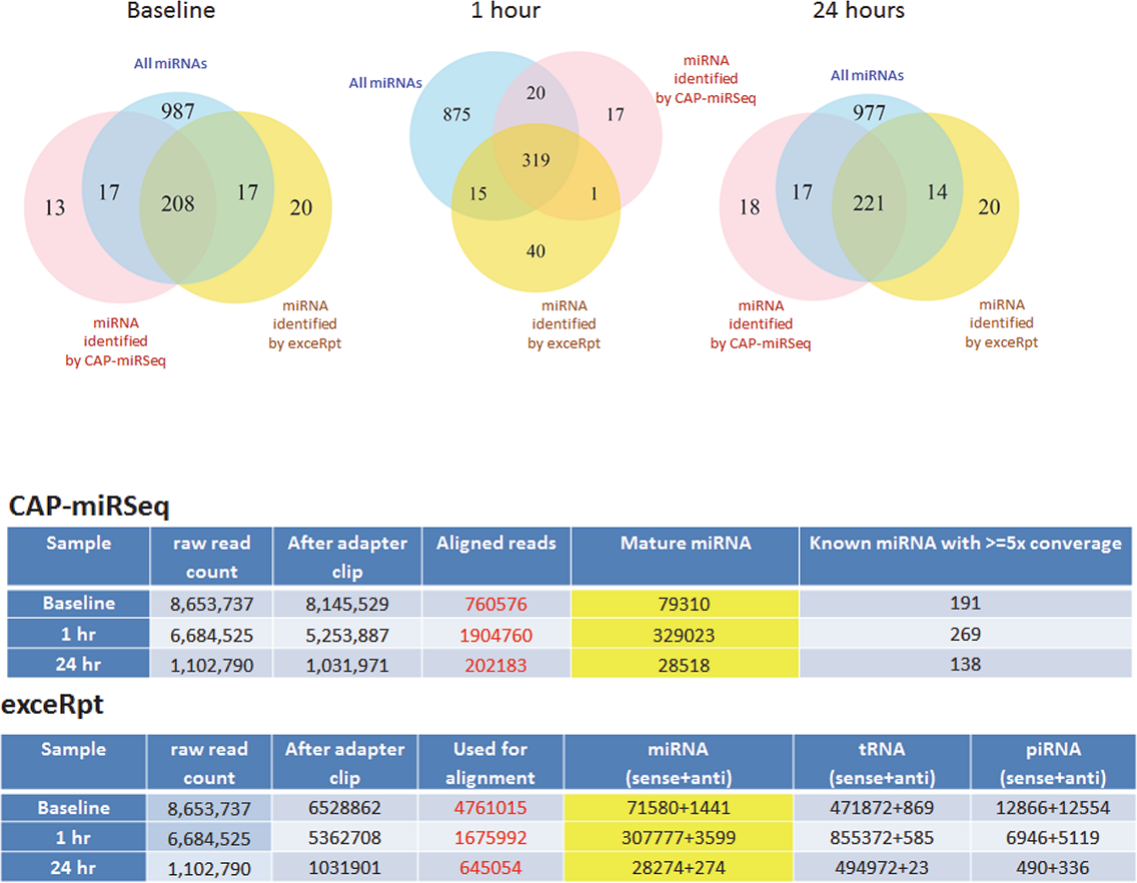
Choice of analytic pipelines for identification of circulating exRNA. (A and B). exRNA samples were obtained at baseline, and at 1 and 24 hours after partial hepatectomy. The number of aligned reads were sufficient for comparison for each sample using the Mayo CAP-miRSeq and Extracellular RNA Communication Consortium exceRpt analytical pipelines. A comparison was made with reference to all 1229 miRNAs that are similar between miRBase versions 11 and 12. (A) The Venn diagrams indicate the number of miRNA identified by each platform compared with all miRNAs. 16.9% of all miRNA were identified in the circulation at baseline by both platforms. This increased to 26.0% at 1 hour and was 18.0% at 24 hours. (B) The exceRpt platform also identified an increase in tRNA at 1 hour, as well as a dramatic reduction in piRNA at 1 and 24 hours compared with baseline.

The discovery approach for circulating extracellular miRNA is based on the analysis of RNA sequencing data and could be dependent on the choice of bioinformatics analytical pipelines. Thus, we directly compared different analytical strategies using data from exRNA samples obtained at baseline, one hour, and 24 hours after hepatectomy. All three samples had sufficient coverage of the miRNAs for comparison of the analytic pipelines. We compared the analyses performed by Maverix Biomics (San Mateo, CA), a Mayo-developed miRNA analytic pipeline (CAP-miRSeq), and the Genboree workbench NIH Extracellular RNA Communications Consortium small RNA pipeline (exceRpt) (S1 Fig, S1 Table). The Maverix Biomics analysis did not provide a read count of miRNA for individual samples, and thus a comparison of read counts for individual miRNA in samples was performed between CAP-miRSeq and exceRpt only. The number of reads mapped to mature miRNA was similar across these platforms. Different versions of miRBase were used (v19 for CAP-miRSeq, v21 for exceRpt), and thus comparison analysis was performed using the 1229 miRNA that were common between miRBase versions 19 and 21. A similar number of miRNAs were identified by both platforms, with an increase in miRNA detected ranging from 16.9% of all miRNA at baseline to 26% at one hour and 18% at 24 hours. The proportion of miRNA within the common set of 1229 miRNA that were detected by only one platform ranged from 1.4–1.6% for the CAP-miRSeq pipeline and 1.1–1.4% for the exceRpt pipeline (Fig 4). However, there were differences in expression of individual miRNAs when analyzed using each platform (S2 Fig). Pair-wise comparison of normalized miRNA counts between the two pipelines revealed excellent correlation for miRNA that had a higher level of expression, whereas at lower expression levels, a greater number of differentially-expressed miRNA were detected using the CAP-miRSeq pipeline (S3 Fig). These observations support the validity of the discovery approach used but additionally emphasize the potential variability that can arise depending on the choice of analytical approach.

## Discussion

Biochemical markers of hepatic function or injury currently used in clinical practice do not provide information on the presence of active regeneration. The availability of such markers would be helpful to guide management when the amount of functional liver is reduced after acute liver injury and necrosis or after partial liver resection. By using an unbiased and comprehensive RNA sequencing-based profiling and data-analysis strategy that included isolation of extracellular RNA, library preparation, RNA sequencing, and RNA annotation, we identified temporal alterations in diverse RNA species within the circulation after partial hepatectomies in C57BL/6 mice. These circulating exRNA may provide a powerful tool for the non-invasive assessment of ongoing liver regeneration. We further (i) identified ncRNA that were both increased in tissue and could be detected in the circulation within extracellular vesicles, (ii) detected novel circulating ncRNA that were increased in the circulation following partial hepatectomy, and (iii) report the utility of a quantitative digital PCR assay with adequate sensitivity for the detection of extracellular RNA that can be used to examine temporal changes in circulating exRNA biomarkers of the regenerative response in the liver.

In this study, selected circulating exRNA that were specifically increased following partial hepatectomy were correlated with temporal data on changes in expression of these RNA within hepatic tissues. An increase in exRNA, such as miRNA, following partial hepatectomy supports their involvement in biological responses. MiRNA expression is altered during the priming phase of liver regeneration, and several genes and proteins are known to have altered expression during this phase. We identified alterations in the hepatic expression of 30 miRNA genes (P < 0.05) within the first 90 minutes after hepatectomy. The alterations in expression of virtually all of these miRNA were transient, consistent with a high degree of regulation and temporal trends in the expression of several factors, such as priming factors at the onset of the regenerative process. The very rapid alterations in circulating extracellular miRNA observed after partial hepatectomy supports their involvement in systemic responses during this early priming phase of the regenerative process. In addition to their potential role as circulating biomarkers of the regenerative response, future studies could examine the mechanistic role of these RNA in modulating gene expression and hepatocyte proliferation during hepatic regeneration, as well as the regulation of their production by progenitor cell populations within the liver.

This analysis presumes that the source of circulating exRNA is remnant hepatic tissue and that enhanced release occurs as a consequence of increased hepatic expression of RNA. Interestingly, changes in the expression of mature miRNA were noted as early as 15 minutes posthepatectomy, with a significant decrease in expression of 23 miRNA by more than 3-fold (P < 0.05). Of note, no appreciable changes were noted in expression levels of the corresponding precursor miRNA, suggesting that the regulation of mature miRNA expression does not involve changes in miRNA transcription or processing from precursor forms. Thus, an increase in exRNA release could involve the selective release of pre-formed RNA or be the result of release by non-hepatic tissues in the absence of increased hepatic expression. Although not evaluated in the present study, disconcordant exRNA candidates for which elevated hepatic expression was not identified may have biological significance or clinical utility.

In addition to known miRNAs, we have identified several other types of ncRNA as candidate markers of ongoing regeneration, such as the novel miRNA and several lncRNA. The biological significance of several of these is unknown. Although the role and involvement of lncRNA in hepatic neoplasia has been extensively explored, the contribution of these RNA to hepatic regeneration or to other physiological or pathophysiological responses in the liver is not well understood. Recent studies have implicated specific lncRNA in hepatic regeneration, and further studies are warranted to systematically examine the biological and functional roles of these candidates. Such studies may offer insights into their contribution to regenerative processes following hepatic resection or small-for-size partial liver transplants.

## Supporting Information

S1 Fig RNA sequencing data analysis pipelines. An overview of two different bioinformatics small RNA sequencing analytic pipelines, CAP-miRSeq (19) and exceRpt (developed by Robert Kitchen at the Gerstein Lab at Yale University and integrated into the Genboree Workbench by Sai Lakshmi Subramanian and William Thistlethwaite at the Bioinformatics Research Laboratory, Baylor College of Medicine, Houston, TX.)
(PPTX)

S2 Fig Heat-map of miRNA expression. Analyses were performed on normalized read counts for miRNA using the exceRpt pipeline (GENBOREE) and the CAP-miRSeq pipeline (Mayo).
(PPTX)

S3 Fig Pair-wise correlations of normalized miRNA counts using two different analytical pipelines. A. Analyses were performed on normalized read counts for all miRNA using the exceRpt pipeline (GENBOREE) and the CAP-miRSeq pipeline (Mayo). B. Analyses was performed for miRNAs with 5 or more raw read counts in at least 1 sample.
(PPTX)

S1 Table A comparison of output data from three different analyses. (PDF)

S2 Table miRNA targets. (PDF)

## Abbreviations

ddPCR: droplet digital PCR
EV: extracellular vesicle
exRNA: extracellular RNA
miRNA: microRNA
VD: vesicle-depleted
lncRNA: long non-coding RNA
ncRNA: non-coding RNA
PH: partial hepatectomy
CT: cycle threshold
