# Supplementary figures and images for: Circulating Extracellular RNA Markers of Liver Regeneration
Source: PLoS One. 2016 Jul 14;11(7):e0155888. doi: 10.1371/journal.pone.0155888 (PMC4945050; doi:10.1371/journal.pone.0155888)

## Slide 1
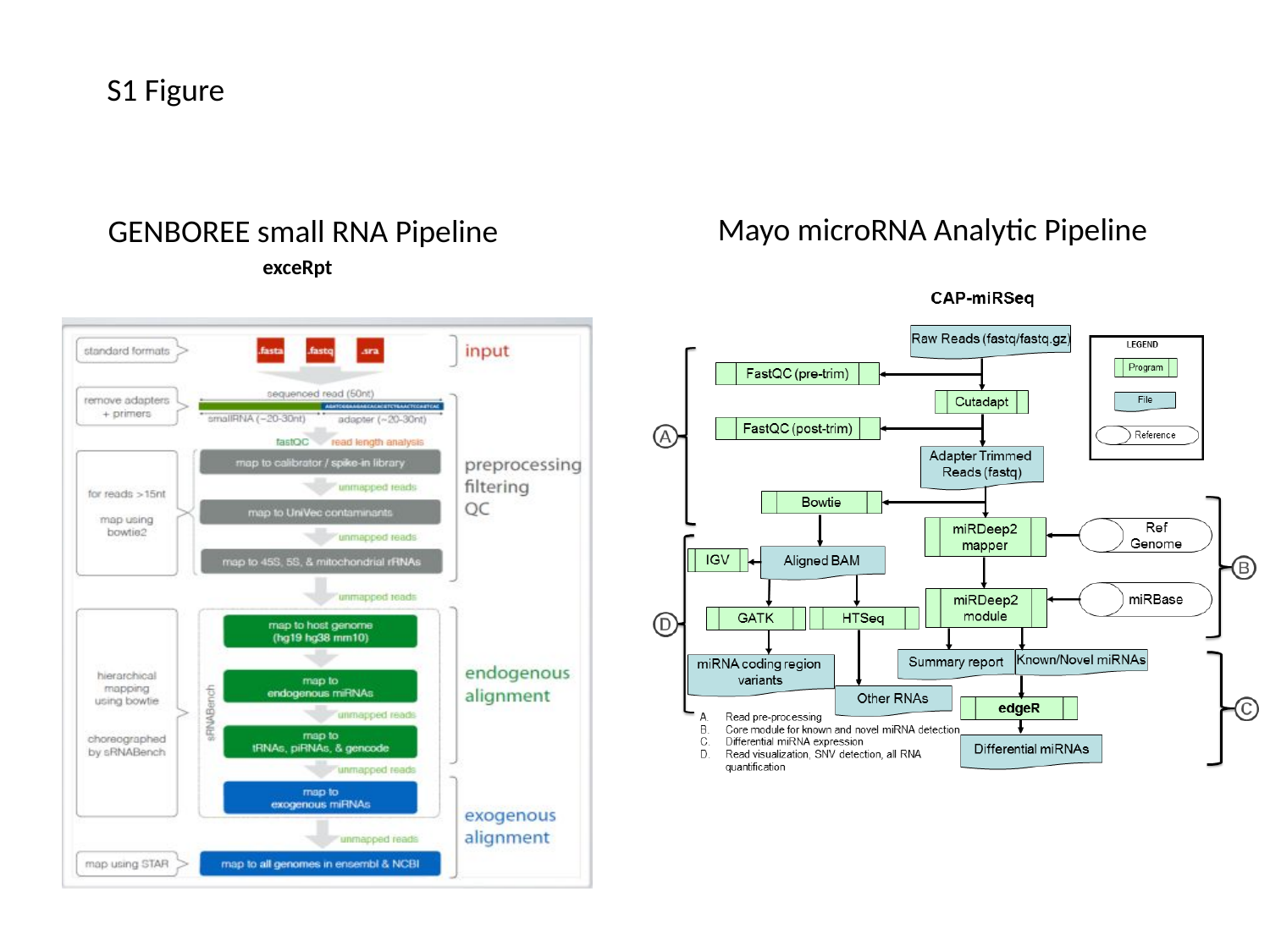

S1 Figure
Mayo microRNA Analytic Pipeline
GENBOREE small RNA Pipeline
exceRpt

Supplement: S1 Fig — An overview of two different bioinformatics small RNA sequencing analytic pipelines, CAP-miRSeq (19) and exceRpt (developed by Robert Kitchen at the Gerstein Lab at Yale University and integrated into the Genboree Workbench by Sai Lakshmi Subramanian and William Thistlethwaite at the Bioinformatics Research Laboratory, Baylor College of Medicine, Houston, TX.) (PPTX) [file pone.0155888.s001.pptx]
