# Supplementary material for: Circulating Extracellular RNA Markers of Liver Regeneration
Source: PLoS One. 2016 Jul 14;11(7):e0155888. doi: 10.1371/journal.pone.0155888 (PMC4945050; doi:10.1371/journal.pone.0155888)
Supplement: S2 Fig — Analyses were performed on normalized read counts for miRNA using the exceRpt pipeline (GENBOREE) and the CAP-miRSeq pipeline (Mayo). (PPTX) [file pone.0155888.s002.pptx]

## Slide 1
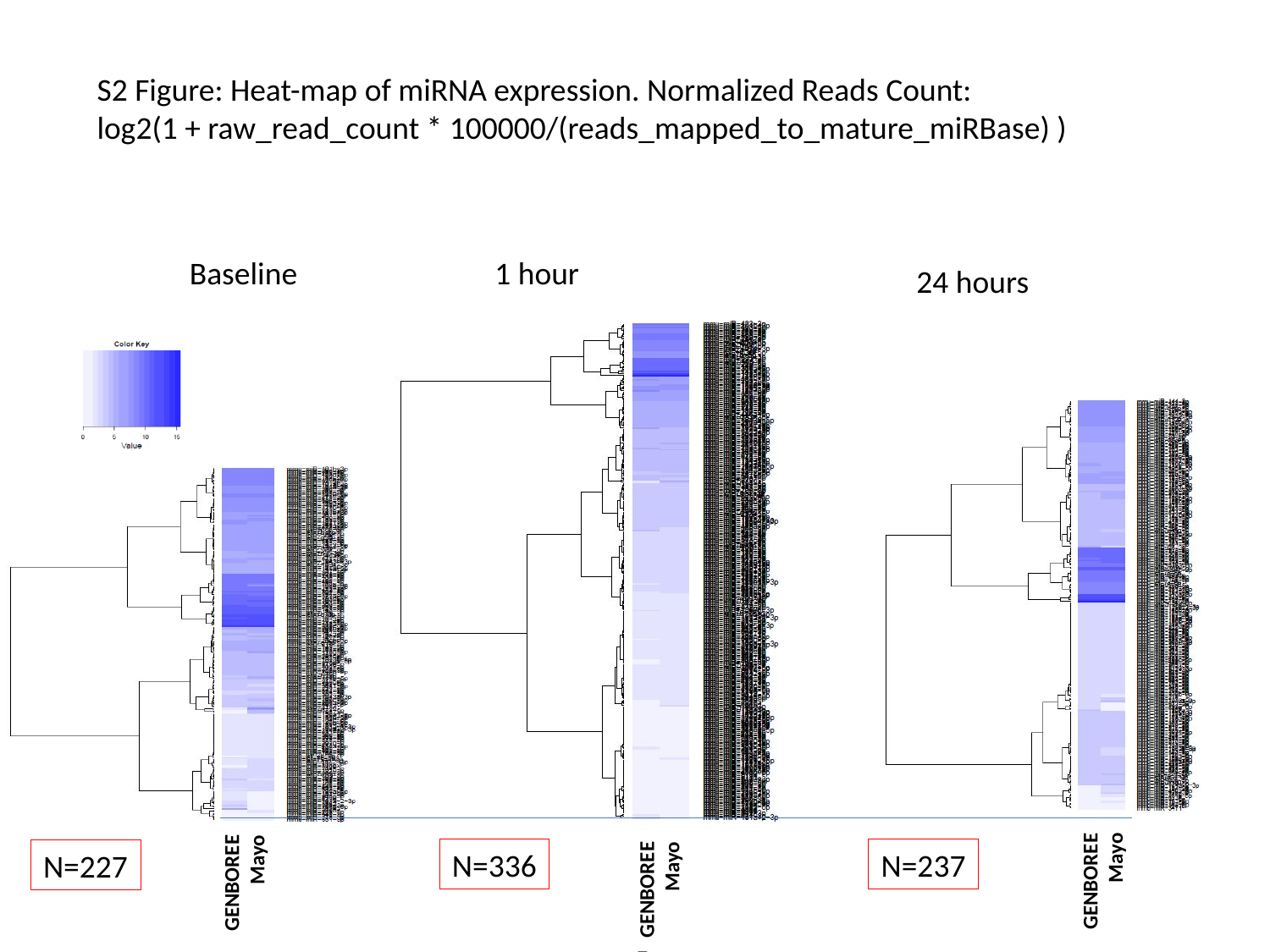

S2 Figure: Heat-map of miRNA expression. Normalized Reads Count:
log2(1 + raw_read_count * 100000/(reads_mapped_to_mature_miRBase) )
Baseline
1 hour
24 hours
N=336
N=237
N=227
GENBOREE
Mayo
GENBOREE
Mayo
GENBOREE
Mayo
