# Supplementary material for: Circulating Extracellular RNA Markers of Liver Regeneration
Source: PLoS One. 2016 Jul 14;11(7):e0155888. doi: 10.1371/journal.pone.0155888 (PMC4945050; doi:10.1371/journal.pone.0155888)
Supplement: S3 Fig — A. Analyses were performed on normalized read counts for all miRNA using the exceRpt pipeline (GENBOREE) and the CAP-miRSeq pipeline (Mayo). B. Analyses was performed for miRNAs with 5 or more raw read counts in at least 1 sample. (PPTX) [file pone.0155888.s003.pptx]

## Slide 1
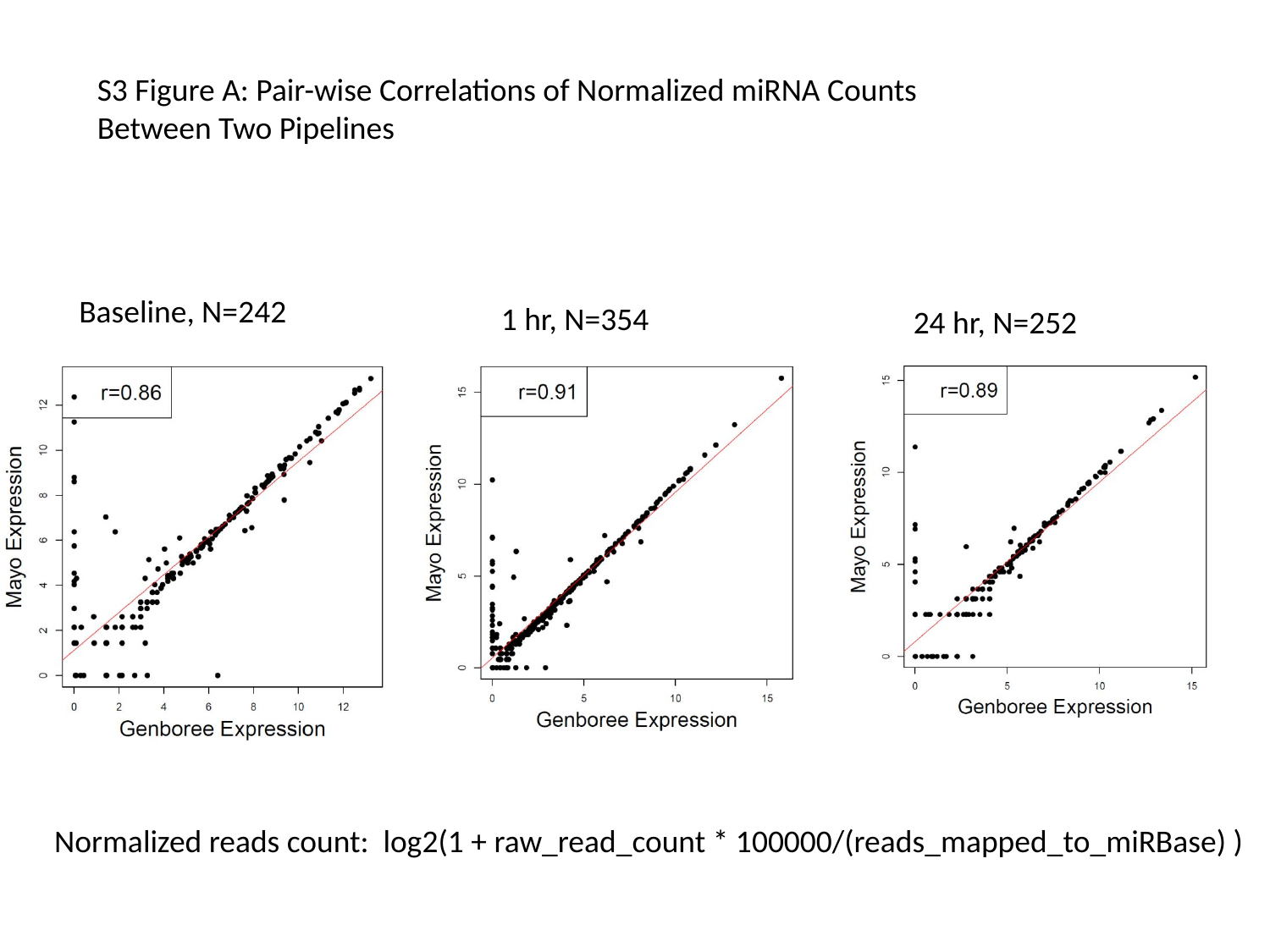

S3 Figure A: Pair-wise Correlations of Normalized miRNA Counts Between Two Pipelines
Baseline, N=242
1 hr, N=354
24 hr, N=252
Normalized reads count: log2(1 + raw_read_count * 100000/(reads_mapped_to_miRBase) )

## Slide 2
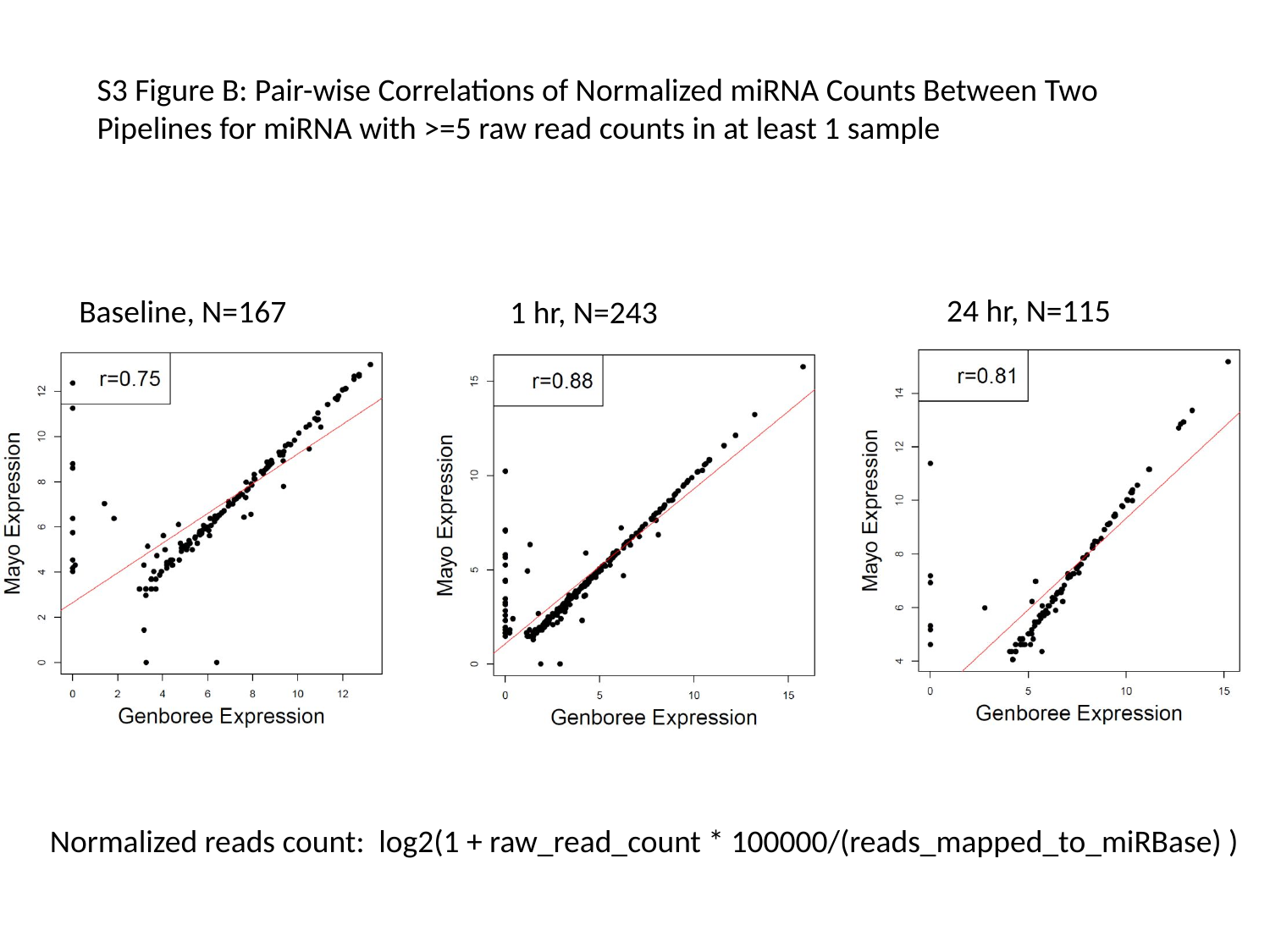

S3 Figure B: Pair-wise Correlations of Normalized miRNA Counts Between Two Pipelines for miRNA with >=5 raw read counts in at least 1 sample
24 hr, N=115
Baseline, N=167
1 hr, N=243
Normalized reads count: log2(1 + raw_read_count * 100000/(reads_mapped_to_miRBase) )
