# Supplementary material for: Circulating Extracellular RNA Markers of Liver Regeneration
Source: PLoS One. 2016 Jul 14;11(7):e0155888. doi: 10.1371/journal.pone.0155888 (PMC4945050; doi:10.1371/journal.pone.0155888)
Supplement: S1 Table — (PDF) [file pone.0155888.s004.pdf]

S1 Table: Comparison of output data

	Maverix	Mayo	Genboree
Read count for mature miRNA		√	√
Possible Novel miRNA		√	√
Differential expressed miRNAs	√		
Variants in miRNAs		√	
Individual Read Mapping Details			√
Exogenous miRNA			√
Ready for IGV view	√	√	
