# Supplementary material for: Circulating Extracellular RNA Markers of Liver Regeneration
Source: PLoS One. 2016 Jul 14;11(7):e0155888. doi: 10.1371/journal.pone.0155888 (PMC4945050; doi:10.1371/journal.pone.0155888)
Supplement: S2 Table — (PDF) [file pone.0155888.s005.pdf]

Supplementary Table 2

targets	description	UniProt
NM_001001182	bromodomain adjacent to zinc finger domain, 2B	Q8CFP4
NM_001001309	integrin alpha 8 (Itga8), mRNA.	A2ARA8
NM_001001321	solute carrier family 35, member D2 (Slc35d2), mRNA.	Q762D5
NM_001003920	BR serine/threonine kinase 1 (Brsk1), mRNA.	Q5RJI5
NM_001004468	transforming, acidic coiled-coil containing protein 2	Q9JJG0
NM_001005508	Rho GTPase activating protein 30 (Arhgap30), mRNA.	Q640N3
NM_001008785	kelch repeat and BTB (POZ) domain containing 8	Q3UQV5
NM_001013022	outer dense fiber of sperm tails 3B (Odf3b), mRNA.	Q5M8M2
NM_001013609	testis expressed gene 24 (Tex24), mRNA.	Q5DP50
NM_001015681	RIKEN cDNA E130308A19 gene (E130308A19Rik), transcript	Q8C4P0
NM_001017426	KDM1 lysine (K)-specific demethylase 6B (Kdm6b), mRNA.	Q8K0Z1
NM_001024945	quiescin Q6 sulfhydryl oxidase 1 (Qsox1), transcript	Q8BND5
NM_001025296	DNA fragmentation factor, alpha subunit (Dffa),	Q8CA98
NM_001029983	mannosidase, alpha, class 1B, member 1 (Man1b1), mRNA.	Q923C1
NM_001033269	eukaryotic translation initiation factor 4E family	-
NM_001033536	regulatory factor X, 7 (Rfx7), mRNA.	Q8CB07
NM_001034037	RIKEN cDNA 1700024G13 gene (1700024G13Rik), mRNA.	-
NM_001034863	transmembrane protein 136 (Tmem136), mRNA.	Q3TYE7
NM_001039088	SEH1-like (S. cerevisiae (Seh1l), transcript variant	Q8R2U0
NM_001039644	ER degradation enhancer, mannosidase alpha-like 3	Q8R1X5
NM_001042592	arrestin domain containing 4 (Arrdc4), transcript	Q9D6S6
NM_001045536	zinc finger, ZZ-type with EF hand domain 1 (Zzef1),	Q5SSH7
NM_001081048	solute carrier family 25 (mitochondrial carrier),	Q9DB41
NM_001081088	low density lipoprotein receptor-related protein 2	Q3V346
NM_001081152	nuclear protein in the AT region (Npat), mRNA.	Q8BPV1
NM_001081205	NIPA-like domain containing 1 (Nipal1), mRNA.	Q8BMW7
NM_001081206	diacylglycerol kinase, iota (Dgki), mRNA.	-
NM_001081366	vacuolar protein sorting 8 homolog (S. cerevisiae)	Q8CIG5
NM_001081417	chromodomain helicase DNA binding protein 7 (Chd7),	A2AJK6
NM_001081433	ankyrin repeat domain 44 (Ankrd44), mRNA.	Q8BYT6
NM_001099296	glycine/arginine rich protein 1 (Grrp1), mRNA.	Q80X91
NM_001102455	amyloid beta (A4) precursor-like protein 2 (Aplp2),	Q9CYS4
NM_001102456	amyloid beta (A4) precursor-like protein 2 (Aplp2),	-
NM_001102670	kelch repeat and BTB (POZ) domain containing 8	-
NM_001109661	BTB and CNC homology 2 (Bach2), transcript variant 1,	Q80WG0
NM_001113180	glutamate receptor, ionotropic, AMPA4 (alpha 4)	Q9Z2W8
NM_001136055	CD82 antigen (Cd82), transcript variant 2, mRNA.	Q3UII2
NM_001139520	SAM domain and HD domain, 1 (Samhd1), transcript	Q8C5C0
NM_001145948	tetratricopeptide repeat domain 39A (Ttc39a),	A2ACP1
NM_001159769	nuclear receptor subfamily 5, group A, member 2	Q99N00
NM_001159986	SEC16 homolog B (S. cerevisiae) (Sec16b), transcript	Q91XT4
NM_001161406	grainyhead-like 1 (Drosophila) (Grhl1), transcript	Q921D9
NM_001162946	pyruvate carboxylase (Pcx), nuclear gene encoding	Q8BP54
NM_001163538	phosphoglycerate mutase family member 5 (Pgam5),	Q8BX10
NM_001164074	TGFB-induced factor homeobox 1 (Tgif1), transcript	Q3TVD4
NM_001164075	TGFB-induced factor homeobox 1 (Tgif1), transcript	-
NM_001164076	TGFB-induced factor homeobox 1 (Tgif1), transcript	-
NM_001164077	TGFB-induced factor homeobox 1 (Tgif1), transcript	-
NM_001164249	tropomyosin 1, alpha (Tpm1), transcript variant 2,	-
NM_001164250	tropomyosin 1, alpha (Tpm1), transcript variant 4,	-
NM_001164252	tropomyosin 1, alpha (Tpm1), transcript variant 6,	-
NM_001164253	tropomyosin 1, alpha (Tpm1), transcript variant 7,	-

NM_001164717	-	-
NM_001165253	-	-
NM_001166495	-	-
NM_001167946	-	-
NM_001170746	-	-
NM_001170855	-	-
NM_001177778	-	-
NM_001190374	-	-
NM_007399	a disintegrin and metallopeptidase domain 10 (Adam10),	Q6NZC0
NM_007405	adenylate cyclase 6 (Adcy6), mRNA.	Q80U07
NM_007489	aryl hydrocarbon receptor nuclear translocator-like	Q9WTL8
NM_007578	calcium channel, voltage-dependent, P/Q type, alpha 1A	Q9R0P8
NM_007594	calumenin (Calu), transcript variant 1, mRNA.	Q8CCN9
NM_007663	cadherin 16 (Cdh16), mRNA.	Q8C730
NM_007729	collagen, type XI, alpha 1 (Col11a1), mRNA.	Q9EQV1
NM_007737	collagen, type V, alpha 2 (Col5a2), mRNA.	Q8BNA3
NM_008018	SH3 and PX domains 2A (Sh3pxd2a), mRNA.	O89032
NM_008143	guanine nucleotide binding protein (G protein), beta	P68040
NM_008580	mitogen-activated protein kinase kinase kinase 5	Q8CC79
NM_008597	matrix Gla protein (Mgp), mRNA.	Q8VEK8
NM_008606	matrix metallopeptidase 11 (Mmp11), mRNA.	Q7TPW7
NM_008695	nidogen 2 (Nid2), mRNA.	Q8R5G0
NM_008702	nemo like kinase (Nlk), mRNA.	Q5SYE6
NM_008711	noggin (Nog), mRNA.	P97466
NM_008822	peroxisomal biogenesis factor 7 (Pex7), transcript	-
NM_008913	protein phosphatase 3, catalytic subunit, alpha	Q8C649
NM_008937	prospero-related homeobox 1 (Prox1), mRNA.	P48437
NM_008967	prostaglandin I receptor (IP) (Ptgir), mRNA.	Q8CC99
NM_009009	RAD21 homolog (S. pombe) (Rad21), mRNA.	Q810A8
NM_009197	solute carrier family 16 (monocarboxylic acid	Q8K3S7
NM_009223	stannin (Snn), mRNA.	Q5M8P0
NM_009330	HNF1 homeobox B (Hnf1b), mRNA.	Q5SS49
NM_009372	TGFB-induced factor homeobox 1 (Tgif1), transcript	-
NM_009436	testis-specific serine kinase 2 (Tssk2), mRNA.	Q6P8M9
NM_009498	vesicle-associated membrane protein 3 (Vamp3), mRNA.	P63024
NM_009520	wingless related MMTV integration site 2b (Wnt2b),	Q544L3
NM_009582	mitogen-activated protein kinase kinase kinase 12	-
NM_009594	c-abl oncogene 1, receptor tyrosine kinase (Abl1),	-
NM_009616	a disintegrin and metallopeptidase domain 19 (meltrin	Q80UF6
NM_009691	amyloid beta (A4) precursor-like protein 2 (Aplp2),	-
NM_009722	ATPase, Ca++ transporting, cardiac muscle, slow twitch	-
NM_009747	bradykinin receptor, beta 2 (Bdkrb2), mRNA.	P32299
NM_009785	calcium channel, voltage-dependent, alpha2/delta	Q9Z1L5
NM_009821	runt related transcription factor 1 (Runx1),	-
NM_009847	CD2-associated protein (Cd2ap), mRNA.	Q9JLQ0
NM_009874	cyclin-dependent kinase 7 (homolog of Xenopus MO15	Q8CAC4
NM_010056	distal-less homeobox 5 (Dlx5), transcript variant 1,	Q810E0
NM_010165	eyes absent 2 homolog (Drosophila) (Eya2), mRNA.	Q811N8
NM_010181	fibrillin 2 (Fbn2), mRNA.	Q61555
NM_010449	homeo box A1 (Hoxa1), mRNA.	Q8BNI8
NM_010469	homeo box D4 (Hoxd4), mRNA.	P10628
NM_010477	heat shock protein 1 (chaperonin) (Hspd1), nuclear	P63038
NM_010513	insulin-like growth factor I receptor (Igf1r), mRNA.	Q60751
NM_010577	integrin alpha 5 (fibronectin receptor alpha) (Itga5),	Q80YP5
NM_010637	Kruppel-like factor 4 (gut) (Klf4), mRNA.	Q60793

NM_010728	lysyl oxidase (Lox), mRNA.	Q3UXV2
NM_010752	mitotic arrest deficient 1-like 1 (Mad1l1), mRNA.	Q9WTX8
NM_010799	multiple inositol polyphosphate histidine phosphatase	Q9Z2L6
NM_010864	myosin VA (Myo5a), mRNA.	Q99104
NM_010942	neuron specific gene family member 1 (Nsg1), mRNA.	Q8C5M2
NM_011122	procollagen-lysine, 2-oxoglutarate 5-dioxygenase 1	Q9R0E2
NM_011204	protein tyrosine phosphatase, non-receptor type 13	Q8CGJ2
NM_011242	RAS, guanyl releasing protein 2 (Rasgrp2), mRNA.	Q9QUG9
NM_011256	phosphatidylinositol transfer protein,	Q6ZPQ6
NM_011322	sodium channel, voltage-gated, type I, beta (Scn1b),	Q8CEH0
NM_011376	single-minded homolog 1 (Drosophila) (Sim1), mRNA.	Q61045
NM_011510	ATP-binding cassette, sub-family C (CFTR/MRP), member	Q9Z2P2
NM_011711	formin-like 3 (Fmnl3), mRNA.	Q8K364
NM_011810	Fas apoptotic inhibitory molecule (Faim), transcript	-
NM_011816	GTPase activating protein (SH3 domain) binding protein	-
NM_011822	phosphatidylinositol glycan anchor biosynthesis, class	Q9QYT7
NM_011829	inosine 5'-phosphate dehydrogenase 1 (Impdh1), mRNA.	Q7TSG7
NM_011892	sarcoglycan, gamma (dystrophin-associated	Q9EQ83
NM_011900	mannose-P-dolichol utilization defect 1 (Mpdu1), mRNA.	Q9R0Q9
NM_011958	origin recognition complex, subunit 4-like (S.	Q9CZF7
NM_011980	zinc finger protein 146 (Zfp146), mRNA.	Q8BQN6
NM_011991	COP9 (constitutive photomorphogenic) homolog, subunit	Q8BX58
NM_013490	choline kinase alpha (Chka), transcript variant 1,	-
NM_013529	glutamine fructose-6-phosphate transaminase 2 (Gfpt2),	Q9Z2Z9
NM_013672	trans-acting transcription factor 1 (Sp1), mRNA.	O89090
NM_013673	nuclear antigen Sp100 (Sp100), mRNA.	Q8C9H3
NM_013683	transporter 1, ATP-binding cassette, sub-family B	-
NM_013693	tumor necrosis factor (Tnf), mRNA.	Q6TDG3
NM_013780	neuronal PAS domain protein 3 (Npas3), mRNA.	Q9QZQ0
NM_013853	ATP-binding cassette, sub-family F (GCN20), member 2	Q99LE6
NM_013890	F-box and WD-40 domain protein 2 (Fbxw2), transcript	Q9QUH1
NM_015734	collagen, type V, alpha 1 (Col5a1), mRNA.	O88207
NM_015760	NADPH oxidase 4 (Nox4), mRNA.	Q9JHI8
NM_015763	lipin 1 (Lpin1), transcript variant 2, mRNA.	-
NM_015801	patatin-like phospholipase domain containing 6	-
NM_016685	cartilage oligomeric matrix protein (Comp), mRNA.	Q9R0G6
NM_016719	growth factor receptor bound protein 14 (Grb14), mRNA.	Q9JLM9
NM_016742	cell division cycle 37 homolog (S. cerevisiae)	Q61081
NM_016866	serine/threonine kinase 39, STE20/SPS1 homolog (yeast)	Q9Z1W9
NM_016889	insulinoma-associated 1 (Insm1), mRNA.	Q63ZV0
NM_016892	copper chaperone for superoxide dismutase (Ccs), mRNA.	Q9WU84
NM_018748	golgi autoantigen, golgin subfamily a, 4 (Golga4),	Q91VW5
NM_018805	heparan sulfate (glucosamine) 3-O-sulfotransferase 3B1	Q9QZS6
NM_018871	tyrosine 3-monooxygenase/tryptophan 5-monooxygenase	Q3TP56
NM_019549	pleckstrin (Plek), mRNA.	Q9JHK5
NM_020008	C-type lectin domain family 7, member a (Clec7a),	Q6QLQ4
NM_020009	mechanistic target of rapamycin (serine/threonine	Q9JLN9
NM_020050	TMEM9 domain family, member B (Tmem9b), mRNA.	Q9JJR8
NM_020329	dolichyl pyrophosphate phosphatase 1 (Dolpp1), mRNA.	Q9JMF7
NM_021310	junction-mediating and regulatory protein (Jmy), mRNA.	Q9QXM1
NM_021314	transforming, acidic coiled-coil containing protein 2	-
NM_021427	family with sequence similarity 181, member B	Q80VF6
NM_021513	THAP domain containing 11 (Thap11), mRNA.	Q9JJD0
NM_021715	carbohydrate (N-acetylglucosamino) sulfotransferase 7	Q9EP78
NM_022325	cathepsin Z (Ctsz), mRNA.	Q9WUU7

NM_024174	mitochondrial ribosomal protein S23 (Mrps23), nuclear	Q99LZ4
NM_024241	kinesin family member 24 (Kif24), mRNA.	Q6NWW5
NM_024427	tropomyosin 1, alpha (Tpm1), transcript variant 3,	-
NM_024435	neurotensin (Nts), mRNA.	Q9D3P9
NM_025280	antigenic determinant of rec-A protein (Kin), mRNA.	Q8K339
NM_025335	transmembrane protein 167 (Tmem167), mRNA.	Q9CR64
NM_025379	cytochrome c oxidase subunit VIIb (Cox7b), nuclear	-
NM_025455	coiled coil domain containing 28B (Ccdc28b), mRNA.	Q9D919
NM_025605	RIKEN cDNA 2400001E08 gene (2400001E08Rik), mRNA.	Q9CQ22
NM_025617	RIKEN cDNA 2210012G02 gene (2210012G02Rik), mRNA.	Q9D3C1
NM_025699	RIKEN cDNA 3230401D17 gene (3230401D17Rik), mRNA.	Q9D722
NM_025705	discoidin, CUB and LCCL domain containing 1 (Dcbld1),	Q9D4J3
NM_025905	tetratricopeptide repeat domain 23 (Ttc23), mRNA.	Q8CHY7
NM_025943	DAZ interacting protein 1 (Dzip1), mRNA.	Q9CPS9
NM_025961	glycine amidinotransferase (L-arginine:glycine	Q9D964
NM_025978	tetratricopeptide repeat domain 14 (Ttc14), transcript	Q9D5Y1
NM_026178	monocyte to macrophage differentiation-associated	Q9CQY7
NM_026267	NECAP endocytosis associated 1 (Necap1), mRNA.	Q9CR95
NM_026386	sorting nexin 2 (Snx2), mRNA.	Q9CWK8
NM_026408	synuclein, alpha interacting protein (synphilin)	Q9D3Y6
NM_026482	ATPase, Ca++ transporting, plasma membrane 1 (Atp2b1),	Q8K314
NM_026622	RIKEN cDNA 3110057O12 gene (3110057O12Rik), mRNA.	Q9CV37
NM_026634	RIKEN cDNA A930018P22 gene (A930018P22Rik), mRNA.	Q9D1Z2
NM_026681	coiled-coil domain containing 88C (Ccdc88c), mRNA.	Q9DCU8
NM_026924	ovo-like 2 (Drosophila) (Ovol2), transcript variant A,	Q8CIV7
NM_027156	DEAD (Asp-Glu-Ala-Asp) box polypeptide 51 (Ddx51),	Q9D6S8
NM_027439	ATPase, H+ transporting, lysosomal accessory protein 2	Q9CYN9
NM_027941	leucine rich repeat containing 34 (Lrrc34), mRNA.	Q9DAM1
NM_028762	RNA binding motif protein 19 (Rbm19), mRNA.	Q8R3C6
NM_028956	pseudouridylate synthase 10 (Pus10), transcript	-
NM_028975	transmembrane protein 33 (Tmem33), transcript variant	Q9CZM3
NM_028979	cytochrome P450, family 2, subfamily j, polypeptide 9	Q9CRQ9
NM_029083	DNA-damage-inducible transcript 4 (Ddit4), mRNA.	Q9D3F7
NM_029211	ring finger protein 121 (Rnf121), mRNA.	Q8R1Z9
NM_029794	cleavage and polyadenylation specific factor 4-like	-
NM_029836	TSPY-like 2 (Tspyl2), mRNA.	Q7TQI8
NM_029851	dynein cytoplasmic 2 heavy chain 1 (Dync2h1), mRNA.	Q45VK7
NM_029868	GC-rich promoter binding protein 1-like 1 (Gpbp1l1),	Q8BHT3
NM_029939	coiled-coil domain containing 151 (Ccdc151),	-
NM_030035	golgi autoantigen, golgin subfamily b, macrogolgin 1	Q9CTU9
NM_030676	nuclear receptor subfamily 5, group A, member 2	-
NM_033354	SEC16 homolog B (S. cerevisiae) (Sec16b), transcript	-
NM_033601	B-cell leukemia/lymphoma 3 (Bcl3), mRNA.	Q9Z2F6
NM_053072	FYVE, RhoGEF and PH domain containing 6 (Fgd6), mRNA.	Q69ZL1
NM_053074	nucleoporin 62 (Nup62), mRNA.	-
NM_080456	mitochondrial ribosomal protein S6 (Mrps6), nuclear	Q3TLQ4
NM_133208	zinc finger protein 287 (Zfp287), mRNA.	Q9EQB9
NM_133234	BCL2 binding component 3 (Bbc3), mRNA.	Q99ML1
NM_133668	solute carrier family 25 (mitochondrial carrier,	Q8VEM8
NM_133743	Ly6/Plaur domain containing 3 (Lypd3), mRNA.	Q91YK8
NM_133788	isoprenylcysteine carboxyl methyltransferase (Icmt),	Q9EQK7
NM_133804	transmembrane protein 132A (Tmem132a), mRNA.	Q922P8
NM_133889	BSD domain containing 1 (Bsdc1), mRNA.	Q80Y55
NM_134033	coiled-coil domain containing 117 (Ccdc117), mRNA.	Q6PB51
NM_134068	dual specificity phosphatase 22 (Dusp22), transcript	-

NM_134090	KDEL (Lys-Asp-Glu-Leu) endoplasmic reticulum protein	Q8R1L4
NM_134117	protein kinase domain containing, cytoplasmic (Pkdcc),	Q5RJI4
NM_138673	stabilin 2 (Stab2), mRNA.	Q8R4U0
NM_144522	TBC1 domain family, member 10b (Tbc1d10b), mRNA.	Q8BHL3
NM_144549	tribbles homolog 1 (Drosophila) (Trib1), mRNA.	Q8K4K4
NM_144551	tribbles homolog 2 (Drosophila) (Trib2), mRNA.	Q8K4K3
NM_144556	leucine-rich repeat LGI family, member 4 (Lgi4), mRNA.	Q8K1S1
NM_144856	solute carrier family 22 (organic anion transporter),	Q91WU2
NM_145382	family with sequence similarity 193, member B	-
NM_145833	lin-28 homolog (C. elegans) (Lin28), mRNA.	Q99MW2
NM_145890	grainyhead-like 1 (Drosophila) (Grhl1), transcript	-
NM_145933	beta galactoside alpha 2,6 sialyltransferase 1	Q9JJM6
NM_145964	cDNA sequence BC002199 (BC002199), mRNA.	Q99LV9
NM_145972	cDNA sequence BC027231 (BC027231), mRNA.	Q8R2U2
NM_145997	lysine (K)-specific demethylase 5A (Kdm5a), mRNA.	Q921T5
NM_146014	cerebral cavernous malformation 2 homolog (human)	Q8K2Y9
NM_146067	Vcalcineurin-like phosphoesterase domain containing 1	Q8BFS6
NM_146099	DNA segment, Chr 19, Wayne State University 162,	Q8BGW2
NM_153178	eukaryotic translation initiation factor 2C, 2	Q8R3Q7
NM_153194	zinc finger protein 740 (Zfp740), mRNA.	Q6NZQ6
NM_153392	tetratricopeptide repeat domain 39A (Ttc39a),	-
NM_153521	leucine rich repeat containing 41 (Lrrc41), mRNA.	Q8K1C9
NM_153786	vestigial like 2 homolog (Drosophila) (Vgll2), mRNA.	Q8BGW8
NM_153800	Rho GTPase activating protein 22 (Arhgap22), mRNA.	Q8BL80
NM_170728	ankyrin 3, epithelial (Ank3), transcript variant 1,	-
NM_172154	ligand dependent nuclear receptor corepressor (Lcor),	Q6ZPI3
NM_172692	glucosidase beta 2 (Gba2), mRNA.	Q69ZF3
NM_172696	InaD-like (Drosophila) (Inadl), transcript variant 1,	-
NM_172704	DnaJ (Hsp40) homolog, subfamily C, member 11	Q5U458
NM_172726	RIKEN cDNA E130309D02 gene (E130309D02Rik), mRNA.	Q8BGA7
NM_172748	F-box and leucine-rich repeat protein 19 (Fbxl19),	Q6PB97
NM_172821	mitogen-activated protein kinase kinase kinase 13	-
NM_172882	WD repeat and FYVE domain containing 3 (Wdfy3), mRNA.	Q8CHB9
NM_172891	serine/threonine/tyrosine kinase 1 (Styk1), mRNA.	Q6J9G1
NM_173350	oxysterol binding protein-like 9 (Osbpl9), transcript	-
NM_173391	tryptophan hydroxylase 2 (Tph2), mRNA.	Q8CGV2
NM_175271	lysophosphatidic acid receptor 4 (Lpar4), mRNA.	Q8BLG2
NM_175312	RIKEN cDNA B630005N14 gene (B630005N14Rik), mRNA.	Q8BXK4
NM_175358	zinc finger, DHHC domain containing 15 (Zdhhc15),	Q8BGJ0
NM_175493	G protein-coupled receptor 68 (Gpr68), mRNA.	Q8BFQ3
NM_175533	RIKEN cDNA 5830411N06 gene (5830411N06Rik), transcript	-
NM_175535	Rho GTPase activating protein 20 (Arhgap20), mRNA.	Q8R0E4
NM_175638	WNK lysine deficient protein kinase 4 (Wnk4), mRNA.	Q8C1P8
NM_177142	lipase, member I (Lipi), mRNA.	Q8BVB7
NM_177231	arrestin, beta 1 (Arrb1), transcript variant a, mRNA.	Q8BWG8
NM_177660	zinc finger and BTB domain containing 10 (Zbtb10),	Q8BNK5
NM_177663	interferon stimulated exonuclease gene 20-like 2	Q8BVS4
NM_177755	kelch-like 38 (Drosophila) (Klhl38), mRNA.	-
NM_177855	mediator of RNA polymerase II transcription, subunit	Q8BQM9
NM_178683	DEP domain containing 1B (Depdc1b), mRNA.	Q8BH88
NM_178685	protocadherin 20 (Pcdh20), mRNA.	Q8R1A3
NM_178692	RIKEN cDNA C130074G19 gene (C130074G19Rik), mRNA.	-
NM_178706	sialic acid binding Ig-like lectin H (Siglech), mRNA.	Q4QQK7
NM_178710	salt inducible kinase 2 (Sik2), mRNA.	Q8CFH6
NM_178802	tripartite motif-containing 65 (Trim65), mRNA.	Q8BJT1

NM_178912	Fanconi anemia, complementation group M (Fancm), mRNA.	Q8CE63
NM_178935	RIKEN cDNA 4932441K18 gene (4932441K18Rik), mRNA.	Q8BHN1
NM_181040	ATP synthase mitochondrial F1 complex assembly factor	Q811I0
NM_181072	myosin IE (Myo1e), mRNA.	Q91ZI4
NM_181073	pleckstrin homology domain containing, family H (with	Q80TI1
NM_181400	WD repeat domain 47 (Wdr47), mRNA.	Q8CGF6
NM_182839	tubulin polymerization promoting protein (Tppp), mRNA.	Q7TQD2
NM_183024	ribonucleoprotein, PTB-binding 2 (Raver2), mRNA.	-
NM_183148	intermediate filament family orphan 2 (Iffo2), mRNA.	Q8R2V2
NM_183162	cDNA sequence BC006779 (BC006779), mRNA.	Q922U3
NM_183173	ankyrin repeat domain 43 (Ankrd43), mRNA.	Q8BLS7
NM_183270	coiled-coil-helix-coiled-coil-helix domain containing	Q8BT51
NM_184053	calumenin (Calu), transcript variant 2, mRNA.	-
NM_194336	macrophage activation 2 like (Mpa2l), mRNA.	Q7TMV8
NM_197945	ProSAPiP1 protein (Prosapip1), mRNA.	Q6PE51
NM_198007	activating signal cointegrator 1 complex subunit 3	-
NM_198095	bone marrow stromal cell antigen 2 (Bst2), mRNA.	Q8R2Q8
NM_198294	tetratricopeptide repeat, ankyrin repeat and	Q8CHA0
NM_198415	creatine kinase, mitochondrial 2 (Ckmt2), nuclear gene	Q6P8J7
NM_198421	ubiquitin specific peptidase 49 (Usp49), mRNA.	Q9R0X0
NM_198614	family with sequence similarity 148, member C	Q5HZI2
NM_198644	zinc finger and AT hook domain containing (Zfat),	-
NM_199056	inositol 1,3,4,5,6-pentakisphosphate 2-kinase (Ippk),	Q6P1C1
NM_199065	SLIT and NTRK-like family, member 1 (Slitrk1), mRNA.	Q810C1
NM_199143	zinc and ring finger 2 (Znrf2), mRNA.	Q71FD5
NM_199322	DOT1-like, histone H3 methyltransferase (S.	Q6XZL8
NM_201355	N-acetyltransferase 14 (Nat14), mRNA.	Q8BVG8
NM_206856	transforming, acidic coiled-coil containing protein 2	-
NM_207204	ninein-like (Ninl), mRNA.	Q6ZQ12
NM_207213	sorting nexin 25 (Snx25), mRNA.	Q7TQF4
NM_207217	integrin alpha FG-GAP repeat containing 3 (Itfg3),	Q8C0Z1
NM_207279	phosphatidylinositol-specific phospholipase C, X	Q8CHS4
NR_028355	steroid receptor RNA activator 1 (Sra1), transcript	-
